# Fast neural population dynamics in primate V1 captured by a genetically-encoded voltage indicator

**DOI:** 10.21203/rs.3.rs-5851261/v1

**Published:** 2025-01-20

**Authors:** Jingyang Zhou, Yuzhi Chen, Matt Whitmire, Pin Kwang Tan, Jimin Wu, Ashok Veeraraghavan, Jacob T. Robinson, Wilson Geisler, Vincent A. Pieribone, Eyal Seidemann

**Affiliations:** 1 Center for Computational Neuroscience, Flatiron Institute, New York, USA.; 2 Center for Perceptual Systems, University of Texas Austin, Austin, USA.; 3 Center for Theoretical and Computational Neuroscience, University of Texas Austin, Austin, USA.; 4 Department of Psychology, University of Texas Austin, Austin, USA.; 5 Department of Neuroscience, University of Texas Austin, Austin, USA.; 6 Department of Bioengineering, Rice University, Houston, Texas, USA.; 7 Department of Electrical and Computer Engineering, Rice University, Houston, Texas, USA.; 8 The Jon B. Pierce Laboratory, New Haven, CT, USA.; 9 Department of Cellular & Molecular Physiology, Yale University, New Haven, CT, USA.; 10 Department of Neuroscience, Yale University, New Haven, CT, USA.; 11 Lamont Doherty Earth Observatory at Columbia University, Palisades, New York, USA.

## Abstract

Genetically encoded voltage indicators (GEVIs) can measure millisecond-scale subthreshold neural responses with cell type specificity. Here, we successfully expressed, for the first time, a GEVI in excitatory V1 neurons in macaque monkeys. We then used widefield fluorescent imaging to measure V1 dynamics in response to visual stimuli with diverse temporal waveforms and contrasts, and compared these responses to signals measured using a genetically encoded calcium indicator (GECI) and a synthetic voltage-sensitive dye (VSD). When compared to GECI, GEVI captures faster response dynamics, tracks higher temporal frequencies, and responds to lower contrasts. To quantitatively characterize these three signals, we developed a simple nonlinear model that predicts the response dynamics to stimuli with arbitrary temporal waveforms and contrasts. Our results are consistent with the hypothesis that GEVI signals reflect the dynamics of locally summed membrane potentials, thus opening the door for a new class of experiments in behaving primates.

## Introduction

Neural computations underlying complex behaviors are implemented in the brain by circuits composed of diverse populations of neurons that interact in complex and nonlinear ways. To understand how such neural computations are implemented in the brain, we need tools that would allow us to measure electrical signals from specific neuronal cell types in real time, in behaving subjects performing complex behavioral tasks. Genetic approaches using viral vectors and specific promoters (or enhancers) provide a powerful tool for expressing genetically encoded activity reporters in selective cell types in a dense and intermixed brain tissue. These genetic methods, when combined with imaging approaches such as widefield, multiphoton or endoscopic imaging, provide a powerful way to study neural activity in behaving animals.

The most commonly used activity reporters for circuit dissection in behaving animals are genetically encoded calcium indicators (GECIs; [1]). While many important discoveries have been made using GECIs, calcium indicators suffer from several limitations. First, the dynamics of calcium concentration in cells are complex and are affected by multiple cellular mechanisms, making the relationship between the calcium signal and the electrical activity of cells (both sub- and supra-threshold) complex and indirect. Second, while neural computations occur on the time scale of milliseconds, calcium signals are slow, limiting the ability to study neural dynamics in real time. Third, GECI signals are likely to be dominated by supra-threshold spiking activity, and thus provide a limited window into the important sub-threshold dynamics of neural computations.

Genetically encoded voltage indicators (GEVIs) address these limitations by measuring fast subthreshold neural responses with cell type specificity. However, until now, functional imaging studies with activity reporters in non-human primates, which are an important animal model for human perception, cognition and motor control, have been limited to the use of GECIs [2–7], and synthetic voltage-sensitive dyes [3, 8–15] which bind indiscriminately to cell membranes and do not provide cell-type specificity.

Here we report results from a new method using the GEVI ‘pAce’ [16] for chronic widefield imaging from populations of excitatory neurons in the primary visual cortex (V1) of behaving macaque monkeys. We used GEVI to measure the nonlinear dynamics of V1 response to visual stimuli of different temporal waveforms and contrast. We then quantitatively compared the GEVI signals to V1 responses to identical visual stimuli measured using a genetically encoded calcium indicator (GECI; [1]) and a synthetic voltage-sensitive dye (VSD; [17]). When compared to GECI signals, GEVI signals capture much faster and more fine-detailed response dynamics, track higher temporal frequencies, and respond reliably to lower stimulus contrasts. Using a simple computational model, we show that our results are consistent with the hypothesis that widefield GEVI signals capture the locally summed subthreshold activity of V1 neurons (similar to synthetic VSD; [18]), while GECI signals capture the locally summed spiking activity [2] that is then temporally blurred. Finally, we show that widefield GEVI imaging can capture V1 dynamics at the mesoscopic spatial scale of orientation columns [19, 20], and that GEVI imaging can be combined with a recently developed imaging technology – miniature lensless flatscope [21] – which allows measuring columnar-scale population responses over a large V1 area in head unrestrained macaques. Overall, these novel techniques open the door for a new class of experiments to study the neural basis of natural and complex behaviors in non-human primates with high temporal precision and cell-type specificity.

## Results

### Widefield GEVI imaging captures fast and nonlinear dynamics of V1 responses

**Figure 1.**
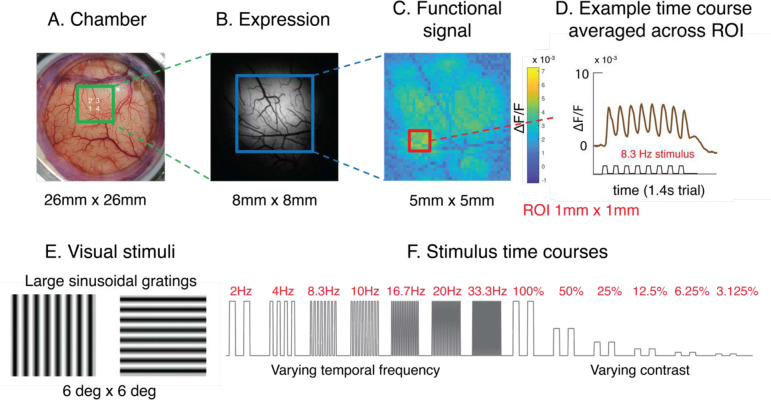
Imaging chamber and visual stimuli. A. Image of one of the optical cranial windows and the recording chamber. The green square is the widefield imaging area centered around a set of four GEVI injection sites. B. Static fluorescent image showing the GEVI expression area. C. Spatial distribution of response to flashed grating at 8.3 Hz. The color indicates response amplitude at 8.3 Hz. D. Example time course from the small 1×1 mm area averaged across 10 trials. E. Visual stimuli were large 6×6 deg^2^ sine wave gratings at two orthogonal orientations. In the temporal series, the timing of a 100% contrast stimulus was varied, while in the contrast series, the contrast of 2Hz stimuli was varied. F. Stimulus dynamics. For varying temporal frequencies, we used 7 frequencies ranging from 2 Hz to 33.3 Hz. For varying contrast conditions, we used 6 stimulus contrasts ranging from 3.125% to 100%.

We used viral vectors (AAVs) to chronically express the GEVI pACE [16] and GECI GCaMP6f [1, 2] in excitatory neurons (using the CaMKIIa promoter) in optical cranial windows over V1 of five macaque monkeys (Fig. 1A–D). One monkey was injected with viruses expressing GEVI and GECI at two separate sites in the same recording chamber, 2 monkeys were injected with GEVI only, and the other 2 monkeys with GECI only (Table 1). To characterize the GEVI response dynamics and spatial resolution, we used widefield fluorescent imaging to measure V1 responses to large (6×6 deg^2^) sine-wave grating stimuli (Fig. 1E) of varying contrast, temporal frequency and orientation (Fig. 1F). In the temporal frequency series, 100% contrast gratings were flashed for 1 second at seven temporal frequencies (2 Hz, 4 Hz, 8.3Hz, 10 Hz, 16.7 Hz, 20 Hz and 33.3 Hz; duty cycle 0.4–0.5). In the contrast series, the contrast of a large grating presented at 2 Hz varied from 3.125% to 100% in six steps (100%, 50%, 25%, 12.5%, 6.25%, and 3.125%). In the orientation series, a 100% contrast grating at six orientations (0 to 150 deg in steps of 30 deg) was presented at 4Hz. In addition, to compare the GEVI dynamics with those obtained with synthetic voltage sensitive dyes [11, 13, 22, 23], we used a VSD (RH1838 or RH1691; [22]) to measure V1 responses to the temporal frequency series stimuli in two of the five macaques. We first focus on analyzing the response time courses within an area of 1 square mm at the center of the expression area (Fig. 1C–D). Analysis of the spatial distribution of the GEVI signals at the scale of the orientation columns, including columnar-scale V1 measurements with a head-mounted lensless camera [21] in a head-unrestrained macaque, will be discussed later.

**Figure 2.**
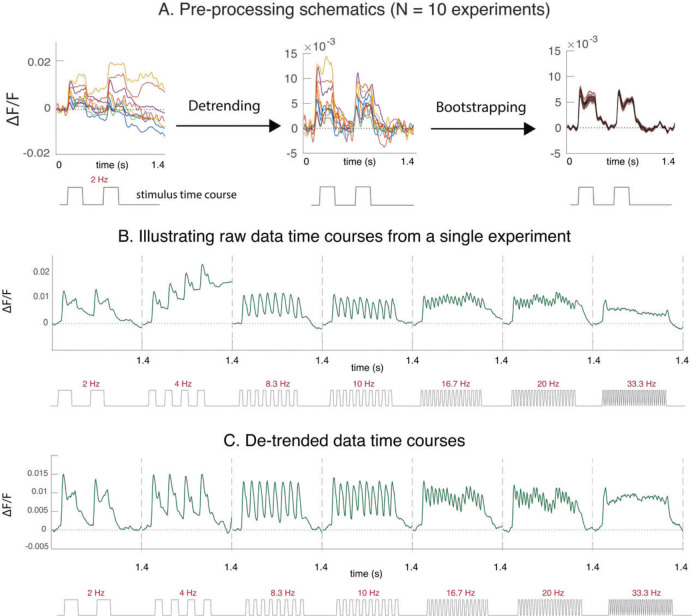
Pre-processing. A. Averaged time courses of GEVI responses to the 2Hz stimulus at one orientation from 10 experiments in 3 monkeys. Each line is the averaged time course across 10 repeats per experiment. B. GEVI response dynamics consist of a fast stimulus-triggered component that is relatively consistent across trials, and a slow component that varies widely from trial to trial. Here, we show Concatenated GEVI temporal dynamics in response to varying stimulus temporal frequencies. Because the slow component is large, averaging across repeats is insufficient to remove this variability. We used a simple and reliable method to separate the fast and slow components [24]. C. We removed the slow component from the response time courses in each experiment (“Detrending”). Finally, we bootstrapped across experiments to assess variability of the fast stimulus-evoked component. Here, we show detrended dynamics from the same experiment as in panel B.

Strong and robust stimulus-dependent dynamics were present in each of the GEVI recording sessions. For example, GEVI signals display clear biphasic dynamics in response to each pulse of the 2 Hz stimuli (Fig. 2A). Each GEVI response time course consists of a fast stimulus-evoked component that reliably tracks the stimulus’ time course, and a slow component that varies across trials (Fig. 2B). The slow component reflects a mixture of neural and non-neural origins. Even though the slow component appears to be stimulus independent, because it is large it cannot be fully eliminated by averaging across repeats. We therefore used a recently developed method to separate the fast stimulus-evoked response from the slow stimulus-independent component [24], and obtained a ‘detrended’ single-experiment time courses (Fig. 2C, see Fig. S1 for more pre-processing details). For the rest of the paper, we focus on the detrended dynamics averaged across experiments.

**Figure 3.**
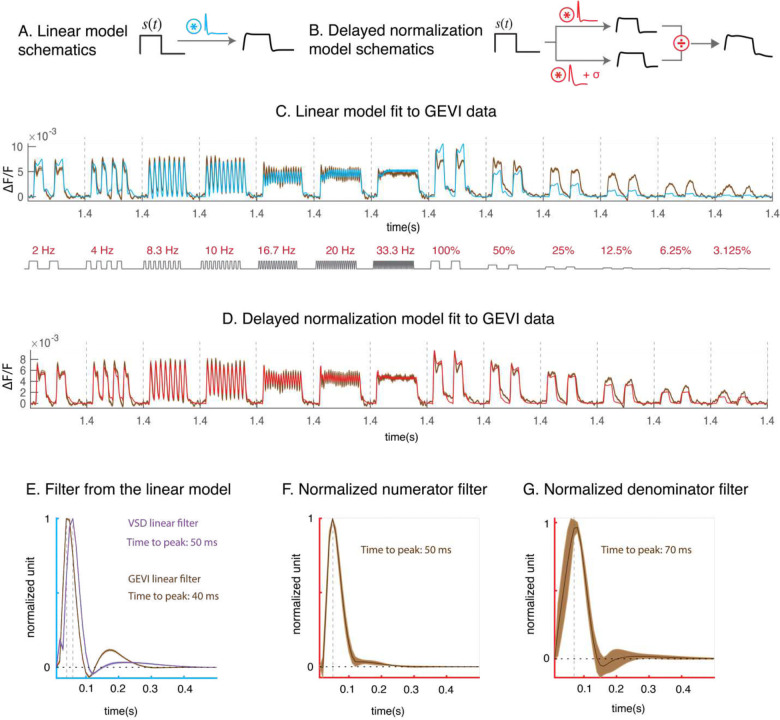
Fitting linear and delayed normalization (DN) model to GEVI dynamics. A. Linear model schematics. A stimulus time course is convolved with a filter to make predictions for the GEVI dynamics. B. DN model schematics. The divisive normalization model consists of two filters (potentially different) convolved with a stimulus time course. The denominator has an additional additive constant taken to prevent the time course from being undefined. C. Linear model fit (blue) to the GEVI averaged dynamics (error bars in the data time course indicates variability across bootstraps). D. Delayed normalization (red) fit to the averaged GEVI dynamics. E. Estimated linear model filters. The estimated GEVI filters are compared to VSD filters (purple) estimated from 6 temporal frequency experiments using 2 monkeys. F. Estimated GEVI filters for the numerator of the DN model. G. Estimated GEVI filters for the denominator of the DN model. In panels F-G, the standard error reflects variability of the filter shapes across bootstraps.

To quantitatively analyze the GEVI dynamics and assess its nonlinearity, we used two models to fit the stimulus-evoked GEVI dynamics – a linear model (Fig. 3A) and a nonlinear delayed normalization model (Fig. 3B)[24, 25]. The linear model consists of a filter f(t) which was fitted to the GEVI data and is convolved with a stimulus time course s(t):

$$r_{l}\left(t\right)=s\left(t\right)\;*\;f\left(t\right).$$

Because, as discussed below, the linear model’s predictions contain systematic biases, we further assessed a nonlinear model – delayed normalization [24, 25]. The delayed normalization model consists of a numerator and a denominator (Fig. 3B). The numerator is similar to the linear model. In the denominator, the stimulus time course is convolved with another filter $f_{n}(t)$, which is typically delayed relative to the numerator filter $f_{l}(t)$ (hence the name “delayed normalization” model). An additional semi-saturation parameter σ is added to the denominator to capture the nonlinearity and prevent the response time course from being undefined when the stimulus contrast is 0. The full delayed normalization model can be described as:

$$r_{n}\left(t\right)=\frac{s\left(t\right)\;*\;f_{l}\left(t\right)}{\sigma \;+\;s\left(t\right)\;*\;f_{n}\left(t\right)}.$$

We fitted both models to the GEVI dynamics averaged across experiments. The linear model largely captures the GEVI response to varying stimulus temporal frequencies, but fails to account for the GEVI response to varying stimulus contrasts. It overestimates the response amplitudes to high contrasts and underestimates the response amplitudes to low contrasts. In addition, the linear model fails to account for the systematic changes in the response time course as a function of contrast (e.g., the change from biphasic to monophasic response, and the increase in latency, with decreasing contrast; Fig. 3C). The delayed normalization model can capture the variations in amplitude and shape of the response dynamics as a function of stimulus contrast (Fig. 3D). The qualitative improvements are also reflected in large quantitative improvement relative to the linear model (R^2^ = 0.933 for delayed normalization model vs. R^2^ = 0.793 for the linear model).

We next examined the shapes of the estimated filters. The linear model filter is tri-phasic (Fig. 3E). This type of filter has commonly been observed in voltage responses of single neurons in early visual stages when nonlinear responses are fitted by a linear model (e.g., [26, 27]). This filter is also similar to the linear filter estimated for the dynamics of population responses in macaque V1 using a synthetic voltage sensitive dye (VSD; Fig. 3E) (see Fig. S2 for VSD preprocessing and Fig. S3 for VSD model fits; [13, 22]).

The estimated numerator and denominator filters of the delayed normalization model (Fig. 3F–G) tend to be simpler in shape (more monophasic) than the linear filter, with the denominator filter having a delayed peak and slower dynamics. These features are qualitatively similar to those observed by fitting the delayed normalization model to V1 response dynamics measured using other methods (electrocorticography or ECoG, VSD, [24]). Overall, our results reveal that widefield GEVI imaging in macaque V1 captures fast nonlinear response dynamics with high fidelity, and that a simple delayed normalization model can quantitatively account for these dynamics.

### Widefield GECI V1 signals are slower and more linear than GEVI signals

Genetically encoded calcium indicators are the most commonly used neural activity reporters (e.g., [1, 2]). Previous work in macaque V1 suggests that widefield GCaMP imaging is dominated by the pooled spiking activity of neural populations [2]. This technique has recently been used to study neural population responses in behaving macaques ([2–4, 21]. To compare the GEVI and GECI responses, we measured GECI responses to an identical set of temporal frequency and contrast stimuli as in the previous section.

**Figure 4.**
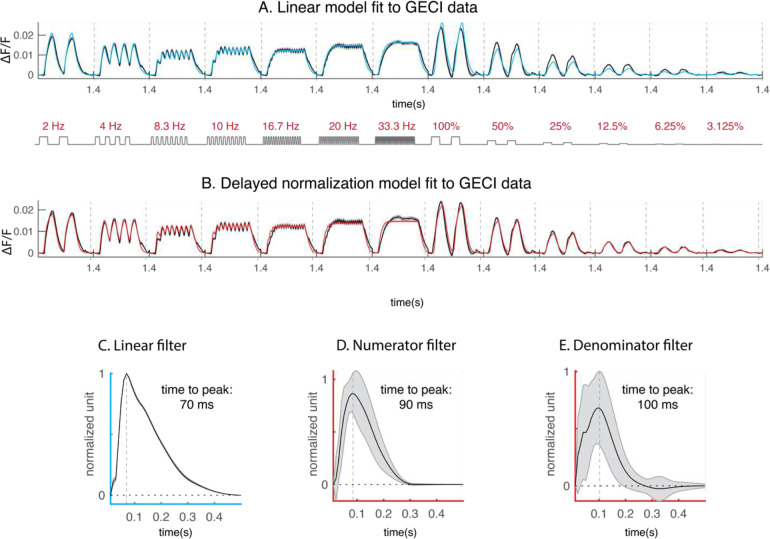
Fitting linear and delayed normalization (DN) model to GECI dynamics. A. linear model prediction to GECI data averaged and bootstrapped across experiments (blue). B. Delayed normalization model predictions (in red) to the GECI dynamics. C-E. Estimated filters for the linear model, and for the numerator and the denominator of the delayed normalization model.

To quantify the GECI dynamics, we fit the same linear and delayed normalization models to the GECI responses (For GECI preprocessing, see Fig. S4). In contrast with the GEVI signal that is strongly nonlinear, the linear model better captures the GECI dynamics, but the delayed normalization model fit shows small but measurable improvements in describing GECI responses to varying stimulus contrasts (Fig. 4A, B). The estimated filter for the GECI linear model, together with the estimated filters for the delayed normalization model all tend to exhibit a monophasic shape (Fig. 4C–E).

**Figure 5.**
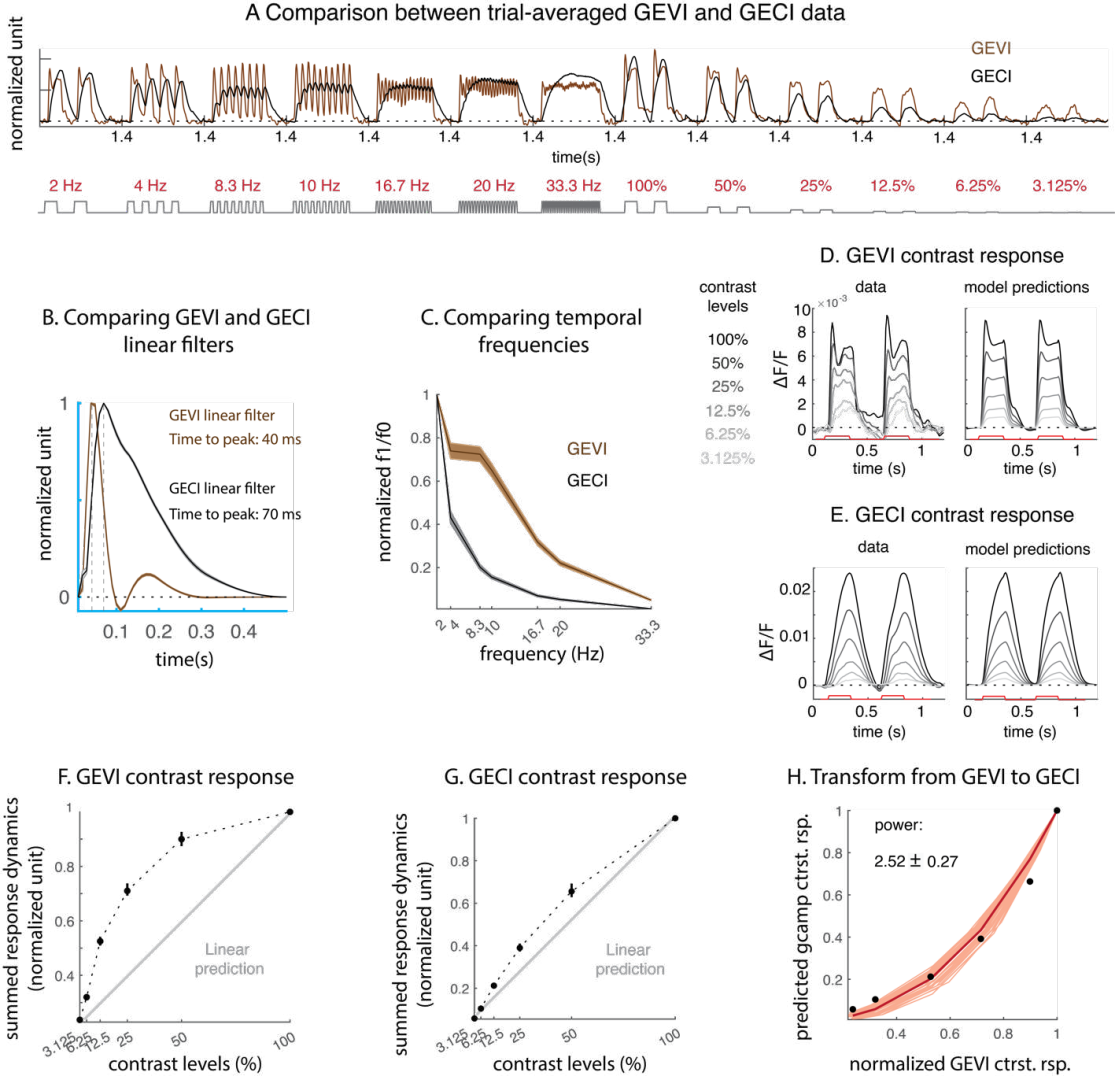
Comparing GEVI and GECI data attributes. A. Comparing averaged GECI (black) and GEVI (brown) dynamics concatenated across stimulus conditions. Each response time course was normalized to its mean. B. Estimated linear filter for GECI (black) and GEVI (brown) data, error bars reflect variability across bootstraps. C. Comparing sensitivity to stimulus temporal frequencies across measurement methods. “f1” is the amplitude of data modulation at the frequency of the stimulus, and “f0” is the overall data amplitude (or the DC). Overall, f1/f0 measures the sensitivity to different temporal frequencies. GEVI has much higher temporal sensitivity than GECI. D. GEVI contrast response dynamics and normalization model predictions. E. GECI contrast response dynamics and normalization model predictions. F. Summed GEVI response dynamics for each contrast condition. Error bars reflect variability across bootstraps. G. Same as F but for GECI responses. H. The power functions that transform each bootstrap of GEVI summed dynamics to GECI summed dynamics. The averaged power is 2.52, with a standard deviation of 0.27.

Compared to GEVI signals, GECI signals display slower dynamics, and vary more linearly with stimulus contrasts (Fig. 5A). The slowness of GECI dynamics can be demonstrated using two analyses. First, the estimated linear GECI filter has a delayed peak relative to the GEVI filter (0.04s for GEVI and 0.07s for GECI) and has much slower decay (Fig. 5B). Additionally, we used spectral analysis to compare GEVI and GECI responses to different stimulus’ temporal frequencies. In Figure 5C, we plot the amplitudes of the response at stimulus frequency (“f1”) divided by the amplitude of the corresponding DC response (“f0”). Large f1/f0 indicates the measurement is sensitive at that stimulus frequency. The GEVI signal is far more sensitive than the GECI signal to high temporal frequencies, and can show clear modulations at temporal frequencies up to and possibly beyond 33.3 Hz.

To compare the GEVI and GECI responses as a function of stimulus contrast, we overlayed each measurement’s response dynamics to different contrast levels on a single panel alongside with the corresponding delayed normalization model predictions (Fig. 5D–E). GEVI and GECI’s contrast response dynamics differ in two ways. First, the shape for the GEVI dynamics for low and high contrast stimuli are drastically different. While the response to high contrast is clearly biphasic, the response to low contrast stimuli is slower and monophasic. In contrast, the GECI response dynamics for low and high contrast stimuli are relatively similar in shape. Similar features can be observed in the delayed normalization model predictions for the two signals. Second, the amplitudes of GEVI and GECI responses exhibit different levels of nonlinearities. This can be observed by examining the summed responses dynamics of each signal as a function of contrast (Fig. 5F–G). For both measurements, the summed response dynamics increase with contrast levels, but the increase in GECI response is slower and more linear with contrast, while the GEVI signal increases faster at low contrast and displays a clearer saturating nonlinearity at higher contrast. Overall, the GECI responses display qualitatively different dynamics than the GEVI responses.

### A linking model between GEVI and GCaMP responses

In previous sections, we summarized GEVI and GECI response dynamics using separate delayed normalization models. Can these two signals be directly related to each other? Here, we test the hypothesis that GEVI and GECI signals are related to each other through simple computations – a static nonlinearity that reflects the transformation from population voltage responses (GEVI) to population spiking activity (GECI), followed by a linear filter that reflects the slow dynamics of intracellular calcium signal and the GECI fluorescence.

To understand the nonlinear transformation between the two measurements, we first examine the nonlinearity in the two measurements’ responses as a function of contrast. The relationship between the summed GEVI and GECI contrast response functions from Figure 5F–G can be summarized using a scaled power function (Fig. 5H). Taking the normalized GEVI contrast response as input, a scaled power function (expansive, with averaged power estimated to be 2.52) transforms these inputs into a prediction for the GECI contrast response. Similar power relationship has been observed in the conversion from membrane potential to spiking activity in single neurons [28–30]) and neural populations [18].

**Figure 6.**
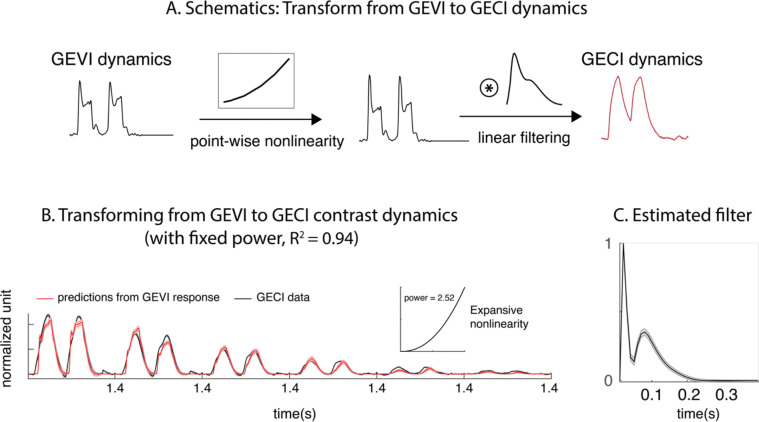
Transform from GEVI to GECI data. A. Schematics of transforming GEVI to GECI contrast dynamics. GEVI dynamics go through a point-wise nonlinearity, parameterized using a power function, before being convolved with a linear filter (the shape of which is to be estimated) to make predictions to the GECI dynamics. B. Averaged GECI contrast dynamics (black), versus the predictions of transformed GEVI contrast dynamics (red). C. The estimated linear filter.

Next, we asked whether applying a linear filter to the GEVI contrast response dynamics following the power function nonlinearity can capture the observed GECI contrast dynamics (Fig. 6A). We estimated a linear filter that would transform the GEVI contrast response dynamics together with a nonlinearity, to predict the GECI signals (Fig. 6B, see Methods). This filter (Fig. 6C) has an interesting biphasic shape. Surprisingly, the combination of a static nonlinearity and a single filter captures the relationship between GEVI and GECI contrast dynamics well (R^2^=0.94). The same operations can be applied to transform GEVI temporal frequency dynamics, together with contrast dynamics, to GECI dynamics as well (Fig. S5).

Overall, our results are consistent with the hypothesis that widefield GEVI signals in macaque V1 reflect the summed membrane potentials while the GECI signals reflect the temporally blurred summed spiking activity, and provide a quantitative model to predict one measure from the other.

### GEVI imaging of orientation maps in head-restrained and head-unrestrained macaque monkey

**Figure 7.**
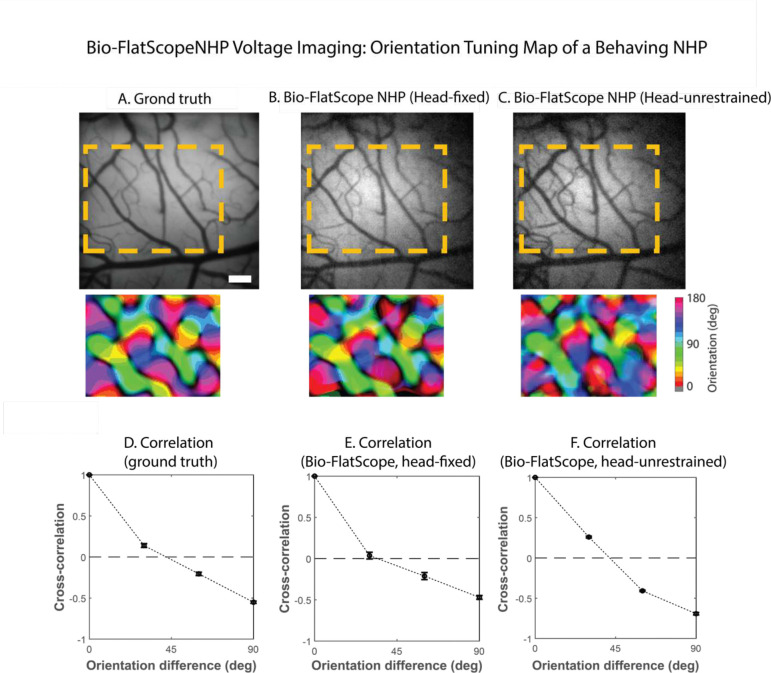
GEVI imaging of V1 orientation columns with epifluorescence microscopy and flatscope. A-C Cortical images taken by epifluorescence microscopy, through a tabletop camera (“ground truth”) with animal head fixed (A), rebuilt from flatscope with animal head fixed (B), and from flatscope with animal head unrestrained (C). Bottom panels show the three corresponding V1 orientation maps calculated from the response maps to 6 orientations by a method of vector sum. The stimuli were large sinusoidal gratings with orientation from 0 deg to 150 deg in step of 30 deg, flashing at 4HZ. The response map to each orientation is created by the Fourier amplitude at 4Hz for each pixel, averaged by 10 trials per orientation. Scale bar in A - 0.5 mm. D-F. Average correlation coefficients between any pair of response maps, collapsed by the orientation difference. Error bars represent the standard errors across all pairs with the same orientation difference.

We next set out to test whether GEVI imaging in macaques V1 has sufficient spatial resolution to provide access to the orientation maps [18, 20]. We further sought to compare the maps obtained with our tabletop widefield imaging system to maps obtained using a recently developed lensless miniscope (Bio-FlatscopeNHP, which we abbreviate as “flatscope” for the rest of the paper; [21]), and compare the maps obtained using the flatscope when the animal’s head is fixed and when the animal head is unrestrained. The results in Fig. 7A show the image of the vasculature and the orientation map obtained from one of the GEVI sites using our tabletop camera. Clear maps with the expected pinwheel structure [19] are observed. To confirm that these maps are not a byproduct of our image pre-processing, we computed the pairwise correlation between the response maps as a function of orientation difference (Fig. 7D). If these maps reflect the organization of orientation columns, the correlation between maps obtained with two orientations should be systematically related to their orientation difference. Consistent with this possibility, maps obtained with orientations that are 30 deg apart tend to be positively correlated, while maps obtained with orthogonal orientations tend to be negatively correlated. This pattern of correlations implies that we are measuring reliable orientation maps. To the best of our knowledge, these are the first orientation maps obtained with GEVI in behaving macaques.

Next, we set out to determine whether we can obtain GEVI-based orientation maps using the flatscope. In the same recording session, we switched from recording with our tabletop camera to the head-mounted flatscope [21]. We then imaged the same patch of cortex while the monkey viewed the flashed gratings at six different orientations. The resulting orientation maps (Fig. 7B) are highly correlated with the maps obtained using the tabletop camera (r^2^ = 0.74). These results show that we can combine these two technologies – GEVI and flatscope imaging - in behaving macaques, and obtain high quality orientation maps while observing the precise dynamics of the response.

Finally, to test whether we can obtain the orientation maps in a head unrestrained macaque, we released the head fixation and performed a head-unrestrained GEVI imaging in macaque V1 (Fig. 7C). While the results are noisier, the map obtained in the head-unrestrained condition is still highly correlated with the map obtained using head fixation (r^2^ = 0.63), demonstrating for the first time that GEVI imaging in head unrestrained macaque V1 can resolve orientation columns.

## Discussion

Non-human primates are an important animal model for human perception, cognition and motor control, but many of the advanced optical and genetic tools available in rodents and simpler organisms are unavailable in non-human primates. Here, we used a novel combination of techniques to optically measure, with cell type specificity, fast neural dynamics in response to a diverse set of visual stimuli by expressing the GEVI ‘pACE’ [16] in excitatory neurons in V1 of behaving macaque monkeys. The GEVI signals display complex and nonlinear dynamics that strongly depend on stimulus contrast. When compared to the responses measured to the same stimuli using a GECI (GCaMP6f; [1]), GEVI captures faster response dynamics, tracks higher stimulus temporal frequencies, and responds to lower stimulus contrasts. We used a simple nonlinear delayed normalization model [24] to separately fit the GEVI and GECI responses. The model allows us to predict the response dynamics to stimuli with arbitrary temporal waveforms and contrasts. We then modeled directly the transfer function from the GEVI to the GECI signals by using a static power-law nonlinearity followed by a temporal blurring filter. Using this GEVI-to-GECI model, we show that our results are consistent with the hypothesis that widefield GEVI signals capture the locally summed subthreshold activity of V1 neurons (similar to synthetic VSDs; [18]), while widefield GECI signals capture the locally summed spiking activity [2] that is then temporal blurred due to the slower intracellular calcium dynamics.

GEVI imaging provides multiple advantages over GECI imaging. It allows a more direct measurement of neural activity, it can track faster temporal dynamics of neural responses, and it provides access to subthreshold neural responses. GEVI imaging also has key advantages over imaging with synthetic VSDs. Synthetic VSDs bind indiscriminately to all membranes, while GEVI can be expressed in specific neural cell types and in specific cellular compartments. In addition, synthetic VSDs require staining the cortex prior to each imaging session, which is challenging and time consuming. VSDs also tend to have strong bleaching during the imaging session, and the quality of the staining degrades over repeated experiments, while GEVI imaging shows little evidence for within-session bleaching and appears to be stably expressed in macaques for extended periods, as with GECI [2]).

The GEVI-measured V1 responses display multiple nonlinearities, consistent with previous electrophysiological and imaging measurements (e.g., [11, 31–33]). First, the dynamics of the response strongly depend on stimulus contrast. While responses to high contrast stimuli are fast and biphasic, responses to low contrast stimuli are delayed and monophasic. Second, the amplitude of the response varies nonlinearly with contrast, displaying accelerating nonlinearity at low contrast and saturating nonlinearity at high contrasts. The changes in the shape of the visual response with contrast are well captured by a delayed normalization model, in which the linear response to the stimulus is modulated divisively by a slower normalization mechanism [24]. While the delayed-normalization model provides a good fit to the observed dynamics, understanding the neural and biophysical mechanisms that underlie this form of nonlinearity await future research.

We find that the transfer function from GEVI signals to GECI signals is well captured by a static nonlinearity (parameterized as a power function; Figs. 5H, 6B), followed by convolution with a biphasic blurring filter (Fig. 6C). The power-law static nonlinearity captures the transform from neural membrane voltage to spiking responses, as has been previously observed in single neurons [34] and neural populations [2, 18]. The linear filter captures the additional dynamics associated with intracellular calcium concentration and with the GECI indicator. The estimated linear filter (Fig. 6C) has an interesting bi-phasic shape, with a fast peak, followed by a decay and then a second delayed peak. This biphasic shape could reflect multiple mechanisms of calcium accumulation in the cell. While the first peak could reflect calcium entry through voltage-gated calcium channels directly associated with spikes, the second peak could reflect slower release of calcium from internal storage through ryanodine receptors [35] or via IP3 receptor by way of metabotropic glutamate receptors [36], or through slower plateau potentials in dendrites [37]. Alternatively, because the widefield signals reflect the pooled activity of a large population of neurons, the two peaks could reflect different calcium dynamics in different subpopulations of V1 neurons and/or neural compartments.

Finally, we show that widefield GEVI imaging in macaque V1 can capture neural dynamics at the mesoscopic spatial scale of orientation columns [19, 20], and that GEVI imaging can be combined with a head-mounted miniature lensless flatscope [21] which allows measuring columnar-scale population responses over a large V1 area in head unrestrained macaques. Overall, the powerful combination of optical and genetic techniques employed here opens the door for a new class of experiments to study the neural basis of complex and more natural behaviors in non-human primates with high temporal precision and cell-type specificity.

## Methods

### Data collection

All procedures have been approved by the University of Texas Institutional Animal Care and Use Committee and conform to NIH standards. Briefly, glass micropipettes were used to inject small volumes (3–5 μL) of rAAV1-CaMKII-pAce (UNC; titer 2.70E+12) or rAAV1-CaMKII-GCaMP6f (Deisseroth lab; titer 4.80E+12) into V1 of 5 macaque monkeys following methods described previously [2]. For the optical imaging in behaving monkeys, a metal head post was implanted for each animal, and a metal recording chamber was placed over the dorsal portion of V1, a region representing the lower contralateral visual field at eccentricities of 2–5 degrees. We imaged GEVI signals from 3 monkeys for the temporal frequency conditions, 2 monkeys for the contrast conditions. For GECI, we imaged two monkeys in the temporal frequency conditions, and two monkeys in the contrast conditions. For VSD, we imaged two monkeys in the temporal frequency conditions. Table 1 summarizes the imaging subjects and the number of experiments.

### Visual stimuli

The stimulus patterns in the temporal and contrast series were 6×6 deg^2^ 2 cycles/deg sinewave grating patterns at two orientations (vertical or horizontal) presented for one second. The animals were presented with 7 temporal frequency conditions. The gratings were presented in square waves of temporal frequency 2, 4, 8.3, 10, 16.7, 20 and 33.3 Hz (duty cycle, 0.4 or 0.5). Subjects were presented with 6 distinct contrast conditions at 2 Hz: 100%, 50%, 25%, 12.5%, 6.25%, and 3.125%. For the orientation series, 100% contrast gratings were presented at 4 Hz at 6 orientations: 0, 30, 60, 90, 120 and 150 deg.

### Data pre-processing

The data processing for GEVI, GECI and VSD is similar to the procedure used in our previous work [24]. In brief, we observed that the time series data in GEVI, GECI and VSD tend to have two components, a fast component that closely tracks the stimulus time course, and a slow component that varies from trial to trial and is largely stimulus independent. The fast component is believed to have a neural origin, and the goal of preprocessing is to extract the fast component from the raw time courses. To do so, we fit a two-component model to the data time course in each experiment. The model, like the data, consists of a fast and a slow component. The fast component is parameterized by a delayed normalization model [24, 25], and the slow component is parameterized by a set of slow basis functions. The pre-processing results are very similar if the fast component is modeled as a filter convolved with the stimulus time courses (a linear model), with the filter being parameterized by a set of fast basis functions [24, 25]. After preprocessing, the fast components from different experiments were averaged across experiments for further analyses.

Other standard data pre-processing steps were taken before the model fitting: (1) Subtracting the averaged time course to blank conditions where no stimulus was presented from the data time series. (2) Aligning the beginning of each response time course (the beginning of each trial) to 0. (3) We shifted response onsets to an earlier time point (30 ms prior) to account for the delay in response onset relative to stimulus onset.

### Bootstrap

To assess the variability of the data and model fits, we bootstrapped response dynamics in the following way. For each stimulus condition, we have multiple recordings across experiments and animals. We re-sampled the recordings (with replacement), and for each set of samples, we computed the averaged data dynamics for each stimulus condition. Then we concatenated these re-sampled averaged dynamics across stimulus conditions, and obtained 50 data time courses (for all stimulus conditions) that were the average across re-sampled experimental repeats. The same bootstrap procedure was applied to GEVI, VSD and GECI analyses.

### Linear model

After de-trending the data, we first fit the linear model to each bootstrap of the data. The linear model consists of a filter $f_{l}(t)$ convolved with the stimulus time course s(t). The filter $f_{l}(t)$ is parameterized as a weighted sum of basis functions $\left\{f_{i}(t)\right\}$. To find the best fit for the linear model is to estimate the weights for these basis functions. The linear model can be represented as:

$$r_{l}(t)=f_{l}(t)\;*\;s(t).$$

The details of the model can be found in [24].

### Delayed normalization model

The delayed normalization model has a divisive form, and consists of a numerator and a denominator. The numerator is a filter $f_{l}(t)$ (parameterized as a weighted sum of basis functions as in the linear model) convolved with the stimulus s(t). The denominator consists of the stimulus time course convolved with a different filter $f_{n}(t)$ that typically has slower dynamics compared to the numerator filter. Additionally, the denominator has an additive constant σ that prevents the model prediction from being un-defined when the stimulus is 0. Overall, the model can be summarized as:

$$r_{n}\left(s\right)=\frac{f_{l}\left(s\right)\;*\;s\left(t\right)}{\sigma \;+\;f_{n}\left(s\right)\;*\;s\left(t\right)}.$$

### Model fitting

For GEVI and GECI data, both the linear and normalization models were fit to the bootstrapped data time courses concatenated across stimulus conditions. For VSD data, both models were fit to bootstrapped time courses concatenated across temporal frequency conditions. The linear model has a linear structure overall, and the weights of the basis functions were found via a closed-form least-square minimization [24]. The parameters of the normalization model were found via a non-linear optimization search [24].

### Contrast response functions

We obtained summed contrast response function for both the GEVI and the GECI data by summing the data time course for each contrast condition. Because there were six contrast conditions, we obtained six numbers for each bootstrap. The same procedure was applied to the GECI data.

### Transforming the contrast response function from GEVI to GECI

We applied a power function ${r_{GEVI}}^{p}$ that transformed the normalized GEVI contrast response function to the normalize GECI contrast response function. The normalization is done so that the response for the 100% contrast condition is 1.

### Predicting GCaMP dynamics using GEVI dynamics

Two steps were involved in predicting the GECI dynamics using the GEVI dynamics: a point-wise non-linearity that was parameterized as a power function, and a linear filter.

We first applied a power function $r_{GEVI}(s{)}^{c}$ to the normalized GEVI responses. Then we applied a filter $f_{t}(t)$ to the transformed GEVI data. The shape of the filter was estimated using linear regression. The entire computation can be summarized as the following:

$$r_{GCaMP}(t)=r_{GEVI}(s{)}^{c}\;*\;f_{t}\left(t\right).$$

We compared the prediction of the GECI dynamics to the observed GECI dynamics.

## Supplementary Material

Supplement 1

## Figures and Tables

**Table 1:** 

	Temporal series	Contrast series
GEVI	M28 x3; M29 x4; M30 x3;	M28 x4; M30 x3;
GCaMP	M31 x7; M30 x3;	M19 x5; M30 x3;
VSD	M28 x1; M29 x5;	
