# Supplementary material for: Fast neural population dynamics in primate V1 captured by a genetically-encoded voltage indicator
Source: Res Sq. 2025 Jan 20:rs.3.rs-5851261. Preprint. [Version 1] doi: 10.21203/rs.3.rs-5851261/v1 (PMC11838743; doi:10.21203/rs.3.rs-5851261/v1)
Supplement: Supplement 1 [file NIHPPrs5851261v1-supplement-1.pdf]

Supplementary Figures

GEVI preprocessing

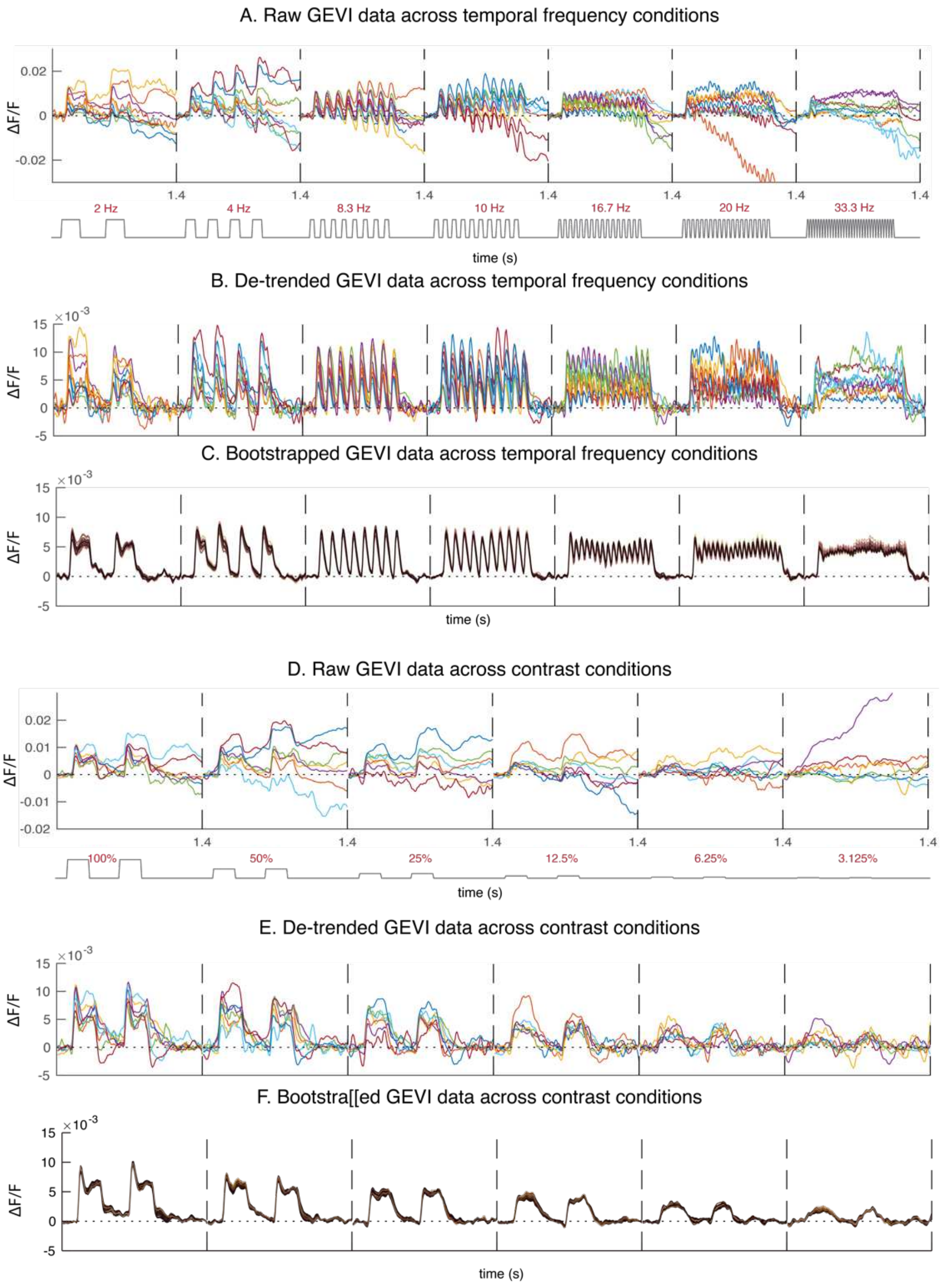

Figure S1. Detailed GEVI preprocessing. A. Ten experimental repeats of GEVI dynamics in response to temporal frequency stimuli. B. Detrended dynamics for the time courses shown in A. C. Bootstrapped dynamics used for further data analyses. D. Experimental repeats of GEVI dynamics in response to varying contrast conditions. E. Detrended dynamics for time courses shown in D. F. Bootstrapped dynamics for further model fitting.

VSD preprocessing

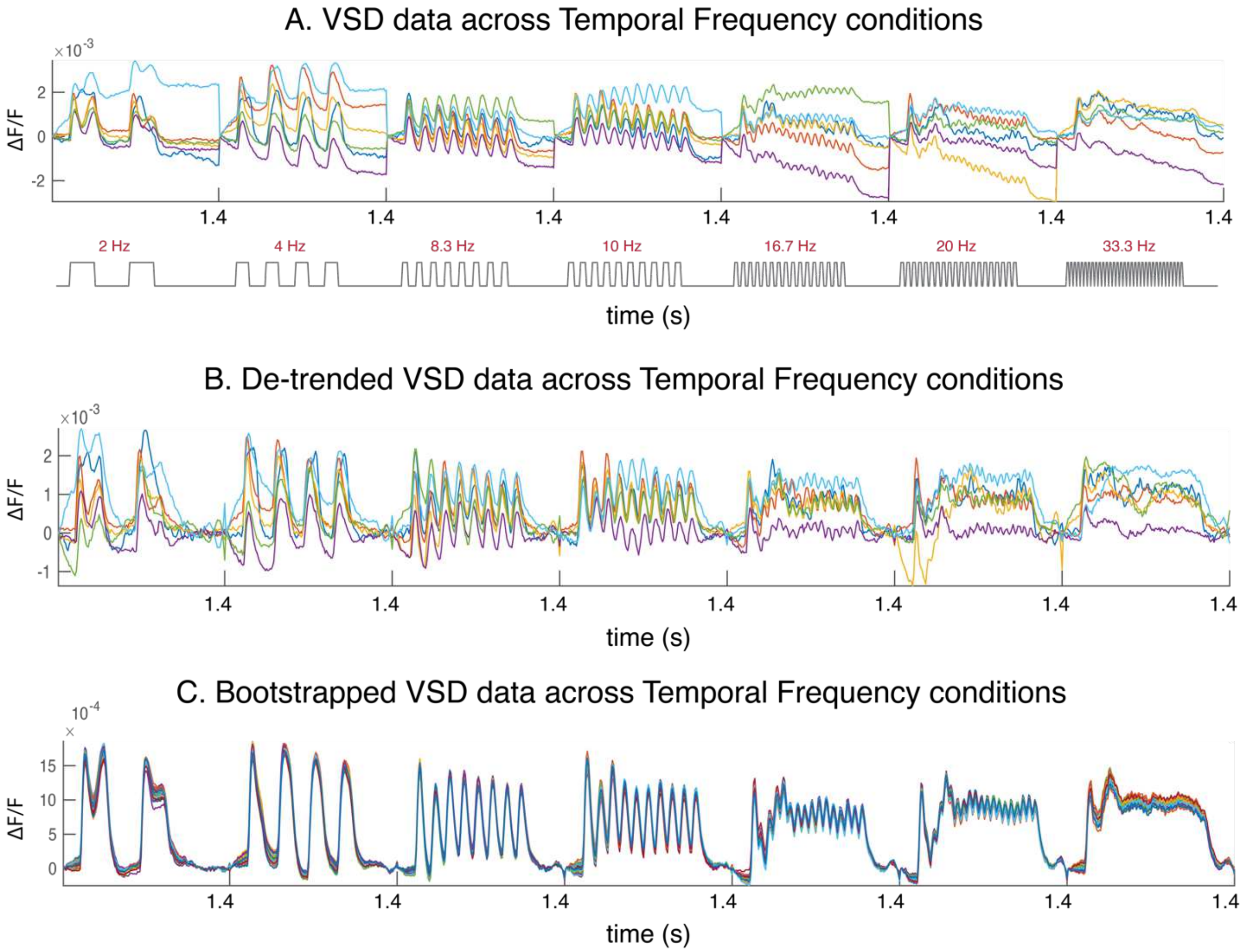

Figure S2. Detailed VSD preprocessing. Same procedure as GEVI pre-processing in Figure S1, except that we only analyzed VSD dynamics to varying temporal frequency conditions.

VSD model fit

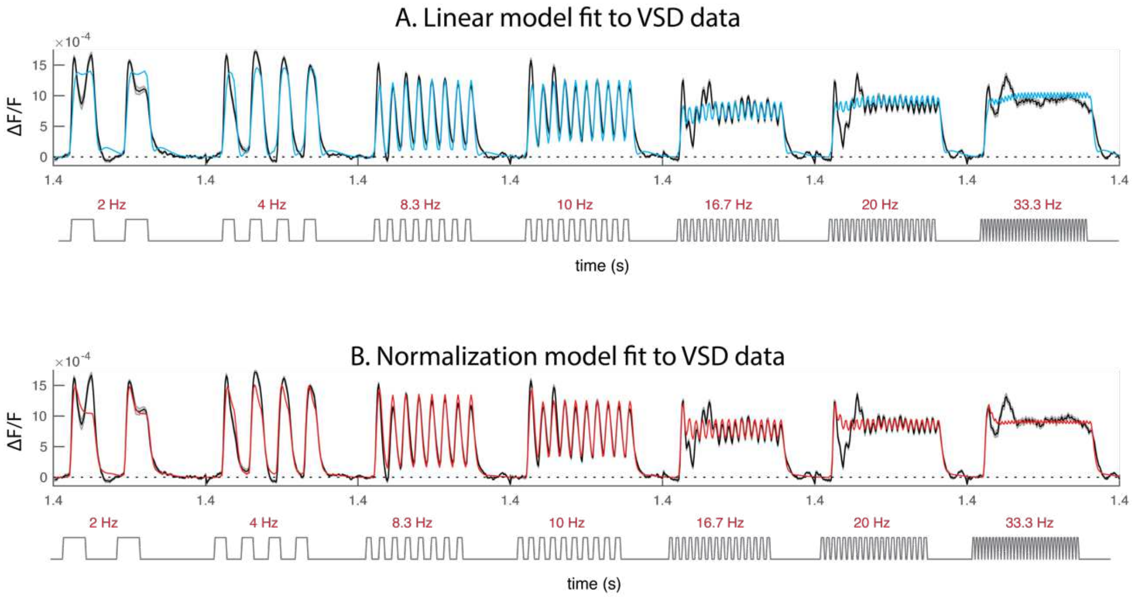

Figure S3. Linear (A) and normalization model fit (B) to VSD data.

GCaMP preprocessing

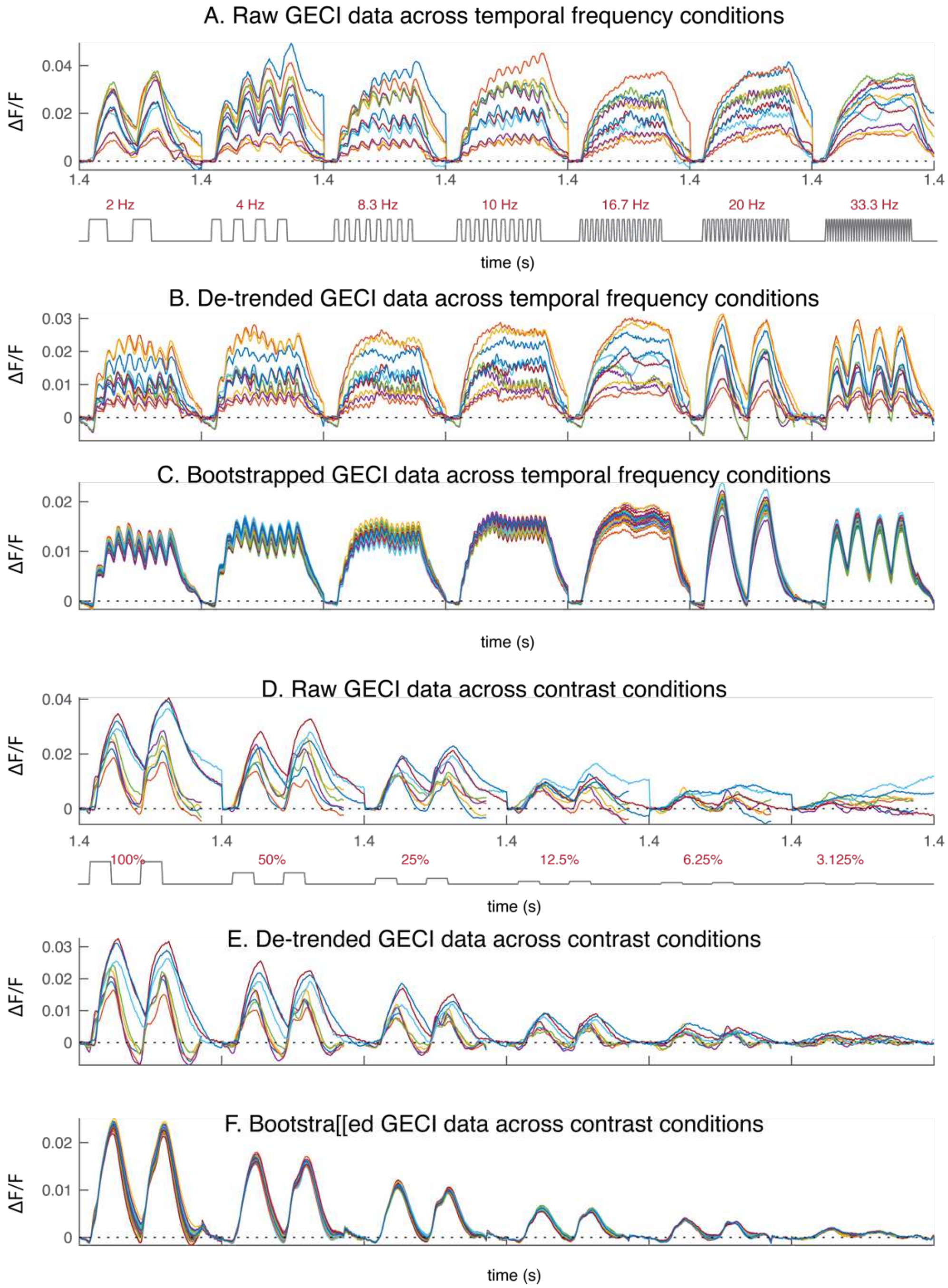

Figure S4. Detailed GECI preprocessing. Same procedure as GEVI pre-processing in Figure S1.

Transforming GEVI to GCaMP data with flexible power

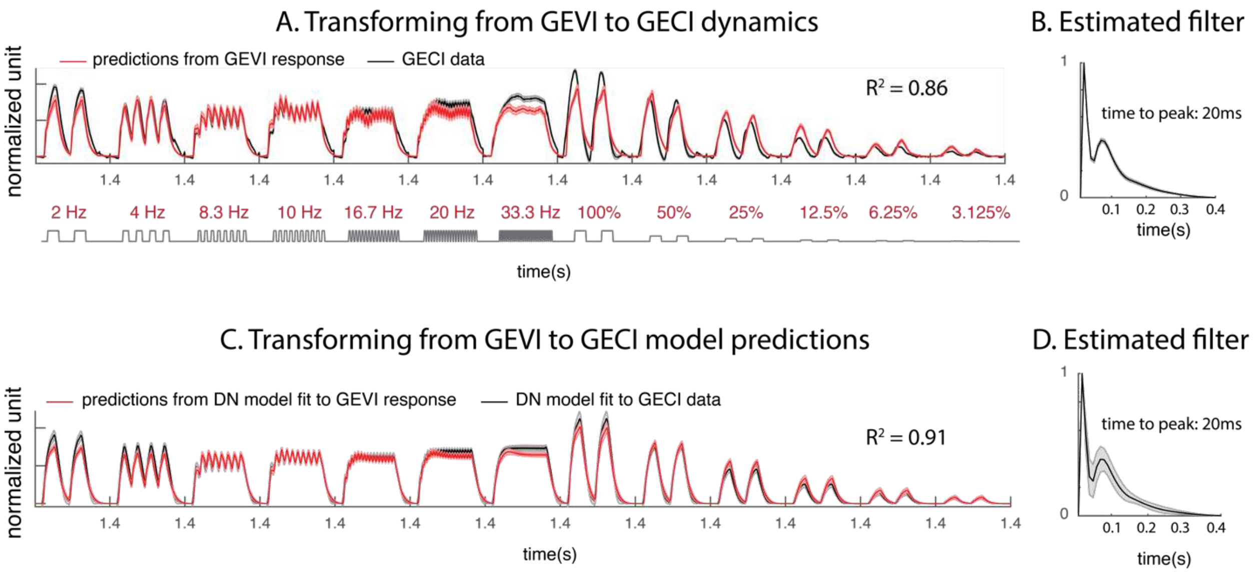

Figure S5. Transforming between GEVI and GCaMP data with flexible power. In Panel A, we demonstrated the prediction of GECI responses (red) by transforming the GEVI data using our linking model. The black shaded line indicates the mean and the standard deviation of the GECI data. B. The filter estimated from the nonlinear transform. The black curve indicates the mean of the estimated filters, and the shaded area indicates the variability of the filter shapes (standard deviation) by fitting different transforms to the bootstrapped data. C. The same analysis as in panel A, but the transform was applied to the DN model prediction of the GEVI responses (red), and the black curve indicates the DN model prediction of the GECI responses. D. Estimated filters from the transform between DN model predictions. Same plotting convention as in panel B.
